# Ovarian torsion in the pediatric population: predictive factors for ovarian-sparing surgery—an international retrospective multicenter study and a systematic review

**DOI:** 10.1007/s00404-022-06522-3

**Published:** 2022-06-25

**Authors:** Claudio Spinelli, Ralf-Bodo Tröbs, Matthias Nissen, Silvia Strambi, Marco Ghionzoli, Alessia Bertocchini, Valentina Cagnetta Domass, Beatrice Sanna, Riccardo Morganti, Francesco Molinaro, Mario Messina, Stefano Tursini, Vito Briganti, Fabrizio Gennari, Gabriele Lisi, Pierluigi Lelli Chiesa

**Affiliations:** 1 Pediatric and Adolescent Surgery Division, Department of Surgical Pathology, Medical, Molecular and Critic Area, University of Pisa, Via Paradisa 2, 56124 Pisa, Italy; 2 Department of Pediatric Surgery, St. Johannes Hospital, Helios Group, Duisburg, Germany; 3 Department of Pediatric Surgery, Marien Hospital, Ruhr-University of Bochum, St. Elisabeth Group, Witten, Germany; 4 Department of Clinical and Experimental Medicine, Section of Statistics, University of Pisa, Pisa, Italy; 5 Division of Pediatric Surgery, Department of Medical, Surgical and Neurological Sciences, University of Siena, Siena, Italy; 6 Department of Pediatric Surgery and Urology Unit, San Camillo Forlanini Hospital, Rome, Italy; 7 Department of Pediatric Surgery, Ospedale Regina Margherita, Torino, Italy; 8 Department of Pediatric Surgery “Spirito Santo”, Hospital of Pescara “G.D’annunzio”, University Chieti-Pescara, Pescara, Italy

**Keywords:** Adolescents, Conservative surgery, Oophorectomy, Ovarian-sparing surgery, Ovarian torsion, Pediatrics

## Abstract

**Study objective:**

Ovarian torsion (OT) in pediatric age is a challenging condition to diagnose and treat. To date, there is still no clear consensus about its management. Our aim was to assess some possible associated factors that can help surgeons in decision-making.

**Design:**

We conducted a retrospective multicentric study of pediatric OT surgically treated between 2010 and 2020 in six Italian and German institutions, comparing our findings with a literature review of the last 10 years (2010–2020).

**Participants:**

Patients aged 0–18 years with a diagnosis of OT intraoperatively confirmed and surgically treated at the involved institutions.

**Results:**

Ninety-seven patients with a mean age at diagnosis of 8.37 years were enrolled in the study. Severe abdominal pain was present in 82 patients (84.5%). Eighty children (82.5%) presented an enlarged ovary with an US diameter > 5 cm and only 32 (40%) of them underwent conservative surgery. A laparoscopic approach was performed in 60 cases (61.9%) although in 15 (15.5%) conversion to open surgery was deemed necessary. A functional cyst was present in 49 patients (50.5%) while 11 children (11.3%) suffered from OT on a normal ovary.

**Conclusions:**

Our results showed that a post-menarchal age (*p* = .001), a pre-operative US ovarian size < 5 cm, (*p* = .001), the presence of severe abdominal pain (*p* = .002), a laparoscopic approach (*p* < .001), and the presence of a functional cyst (*p* = .002) were significantly associated with conservative surgery.

## Introduction

Ovarian torsion (OT) is a rare surgical emergency, which consists in the twisting of the ovarian pedicle around the mesovarium, with consequent impairment of the blood flow, that causes hemorrhagic infarction and necrosis [1]. The incidence of OT in the pediatric population is reported to range from 4.9/100.000 to 20–30/100.000, with an average age of 13 years [2]. Whenever the twisting phenomenon also involves the fallopian tube, we talk about adnexal torsion [3]. The pathological mechanism of OT can be explained by various factors, such as sudden intraabdominal pressure-changes, tubal spasms, hypermobile elongated fallopian tubes and ligaments, with the role of enhanced hormonal activity in the premenarchal and perinatal period still being under discussion [2]. As the clinical presentation is very unspecific and patients often present late, the diagnosis of OT remains challenging and still today based on abdomino-pelvic ultrasound in the first instance, with the exact diagnosis being confirmed only intraoperatively. The differential diagnosis has to consider appendicitis, hemorrhagic or ruptured ovarian cyst, gastroenteritis, ectopic pregnancy, pelvic inflammatory disease and renal colic [4]. The first choice of therapy in case of OT should always be conservative ovarian-sparing surgery, intended as detorsion of the twisted ovary, performed with a laparoscopic approach if possible, followed by the isolated resection of the underlying cyst or tumor if necessary [3]. This article aimed to define factors associated with an ovarian-sparing surgery. After having assessed which preoperative and intraoperative features are mainly connected with OT in young girls, we analyzed a possible association between these and conservative surgery. Furthermore, we decided to perform a systematic review of the studies of pediatric OT published in the last 10 years to verify whether results suggested from our multicentric experience were confirmed by recent literature.

## Materials and methods

This retrospective study included all pediatric (0–18 years old) cases with a diagnosis of OT confirmed intraoperatively, surgically treated from January 2010 to December 2020 at six Italian and German pediatric surgery centers. The contribution for each country grouped into consortiums was 24 cases (24.7%) for Germany (Duisburg, Witten) and 73 cases (75.3%) for Italy (Pisa, Siena, Rome, Pescara). Patients who underwent surgery were all treated by an attending pediatric surgeon (surgical experience > 5 years) with specific skills both in laparoscopy and open surgery assisted by either a trainee or another attending surgeon. Moreover every surgeon involved in this study had specific awareness in attempting to perform conservative surgery: when facing an OT, proceeded to untwist the ovary, waited and see for its vascular recovery and not remove it unless frankly necrotic. Exclusion criteria were age > 18 years, absence of torsion at time of surgery and an intra-operatively revealed alternative diagnosis.

Data regarding patients’ age at time of surgery, menstrual history, side of the affected ovary, presented symptoms, duration of pain, severity of pain, clinical examination, ultrasound examination with ovarian size, surgical intervention, levels of tumor markers (β-HCG, αFP, CA-125, CEA) and histological diagnosis were collected and analyzed.

Patients were divided in pre- and post-menarchal based on the reported menstrual history; when this anamnestic datum was unavailable, patients < 12 years old were classified as premenarchal, and those >12 years of age as post-menarchal, based on the assumption that the age of 12 is the median age at which girls generally have their first menstruation [5]. Regarding the duration of pain, patients were divided into two groups, according to the recent literature [6]: the first group consisted of children who reported a time of onset of the symptoms shorter than 24 h, while the second group was composed of girls who reported a time of onset of the symptoms longer than 24 h before admission. Pain was reported using NRS scale from 0 (no pain) to 10 (worst pain ever) and pain severity was then classified as mild (score 1–3), moderate (4–6) and severe (7–10) [7]. Regarding the size of the twisted ovary seen on the transabdominal ultrasound (US), in accordance with the literature [8], we considered a 5 cm diameter as cut-off for a significant adnexal enlargement.

The nature of the pathology underlying the OT was acquired by definitive histological analysis. Additionally, intraoperative frozen section was performed during surgery in those patients with an intraoperative suspicion of malignancy or to verify the presence of necrosis before practicing oophorectomy. Surgical procedures were led by laparoscopic or open approach and were performed using either the conservative ovarian-sparing treatment or oophorectomy. A conservative ovarian-sparing treatment was defined as detorsion alone or detorsion followed by subsequent cystectomy or tumorectomy, with ovarian preservation and eventually oophoropexy, while non-sparing therapy consisted in oophorectomy [3].

### Data analysis

Categorical data were described using absolute and relative (%) frequencies. To compare the type of treatment with the preoperative and intraoperative features related to OT a Chi-square test was applied. Furthermore, multivariate binary logistic regression was performed to analyze the factors resulted significant by Chi-square test. Significance was fixed at 0.005. All analyses were carried out using SPSS technology version 27.

### Literature review

One author performed a systematic literature review using PubMed database and ClinicalTrials.gov to research articles published from 1st January 2010 to 1st December 2020, responding to the entry “pediatric/children/adolescent ovarian torsion”. Inclusion criteria were full text published in English, patient’s age 0–18 years and information about the surgical treatment (at least if oophorectomy or conservative surgery) available; case reports, systematic reviews and studies on animals were a priori excluded from the review. The assessment of the articles was performed according to the PRISMA flowchart. The eligible studies were analyzed for several features, such as patient’s age (median), right-left side (number of cases), surgical details (number of cases treated with laparoscopic or open approach; number of cases treated with conservative surgery or oophorectomy; number of oophoropexy procedures) and histopathological results. Data were manually collected from reports and recorded in a database. Two different authors independently reviewed all the enrolled studies and the collected reports, in order to reduce reporting bias. This systematic review was not registered and was not financially supported. All the review authors had no competing interests to declare.

## Results

In our group of 97 patients, the average age at the time of operation was 8.37 (range 0–17). Sixty-four girls (66%) were classified as pre-menarchal, while 33 (34%) girls as post-menarchal. Considering all the 97 patients, 49 girls (50.5%) underwent oophorectomy, whereas 48 (49.5%) were treated with conservative ovarian-sparing surgery. Twenty-four (72.7%) of the 33 post-menarchal girls underwent ovarian-sparing surgery, while 40 (62.5%) of the 64 pre-menarchal girls underwent oophorectomy. Laparoscopic surgery was performed in 45 cases (46.4%), open surgery in 37 cases (38.1%) and 15 cases (15.5%) were converted from laparoscopic to open surgery. Data regarding the side of the affected ovary showed an involvement of the right ovary in 53 cases (54.6%), while the left side was involved in 42 patients (43.3%) and two patients had a synchronous bilateral torsion (2.1%). Eighty girls (82.5%) presented an enlarged ovary with a diameter > 5 cm, while only 17 (17.5%) had an adnexal size < 5 cm. Sixteen (94.1%) out of the 17 girls who had an ovarian diameter < 5 cm underwent conservative surgery; on the other hand, in the group of patients with an ovarian size > 5 cm only 32 (40%) underwent conservative ovarian-sparing surgery. Free fluid in the pouch of Douglas was present in 28 cases (28.9%) and preoperative color Doppler ultrasonography examination revealed decreased or absent blood flow to the involved ovary in 62 cases (63.9%). The results of the histopathological examination found that a functional cyst was present in 49 patients (50.5%): these were follicular cysts in 17 cases (34.7%), simple cysts in 15 cases (30.6%), corpus luteum cysts in 7 cases (14.3%), serous cysts in 4 cases (8.2%), pseudocysts in 3 cases (6.2%), fimbrial cyst in one case (2%), paraovarian cyst in one case (2%) and tubarian cyst in one case (2%). Twenty-five patients (25.8%) presented a mature teratoma, six patients (6.2%) were diagnosed with a hemorrhagic corpus luteum, three patients (3.1%) had a cystadenoma, three girls (3.1%) presented a malign tumor, two of these were an immature teratoma and one was a germ cell tumor; 11 children (11.3%) suffered from torsion on a normal ovary: three of them (27.3%) underwent oophorectomy because of the presence of anomalies, such as hemorrhagic infarction and necrotic areas, that were confirmed at the intraoperative frozen section analysis.

Regarding the clinical presentation, abdominal pain was described as severe in 82 patients (84.5%) and as moderate or mild in 15 patients (15.5%).

In the group of children who described abdominal pain as severe, 46 (56.1%) were treated conservatively and 36 (43.9%) with oophorectomy, while in the group of girls who presented mild or moderate abdominal pain, 13 (86.7%) underwent oophorectomy and two (13.3%) conservative surgery. Regarding pain duration, 46 patients (47.4%) reported onset of the symptoms within 24 h from admission, while the other 51 patients (52.6%) had pain for longer than 24 h before admission. Among the first group, 22 (47.8%) experienced oophorectomy and 24 cases (52.2%) underwent ovarian-sparing surgery, while in the second one 27 cases (52.9%) underwent oophorectomy, and 24 cases (47.1%) had a conservative surgery. Tumor markers were available only in 51 cases (52.6%) and were increased in only five patients (9.8%): three girls with a mature teratoma presented an elevation of the Ca-125, one girl with a mature teratoma had increased levels of CEA and the last patient, who reported very high levels of αFP (224 ng/ml), was diagnosed with a mixed germ cell tumor. Of the five children with elevated tumor markers, four underwent oophorectomy and one conservative surgery, whereas, among the children with normal ranged tumor markers, 26 (56.5%) underwent conservative surgery and 20 (43.5%) oophorectomy. Oophoropexy was performed in only one case (1%). Table 1 shows the statistical analysis performed on all the parameters we investigated in our cohort of patients: age < 12 years associated with oophorectomy whereas age > 12 years correlated with conservative surgery (*p* = 0.001), US diameter > 5 cm related to oophorectomy whilst US diameter < 5 cm more associated with conservative surgery (0.001); mild/moderate pain correlated with oophorectomy (0.002), laparoscopy was related to conservative surgery and open surgical approach associated with oophorectomy (*p* < 0.001). Age, US diameter, pain and surgical approach resulted significant following a multivariate binary logistic regression.

**Table 1:** Literature review: articles published between 2010 and 2020

Author	Journal	Year	N° of patients	Median age (mean age)	Side^a^	Underlying pathology	Treatment^b^	Pexy	Approach^c^
Julania et al. [9]	J Minim Invasive Gynecol	2020	54	(9.80 ± 3.95)	–	–	52—CONS 2—OO	–	Lap—53 Open—1
Geimanaite et al. [10]	J Pediatr Surgery	2019	39 (42 ovaries)	(10.48 ± 5.64)	–	15—cyst 1—teratoma 26—not specified	42—CONS	3	Lap—37 Open—5
Tasset et al. [11]	J Pediatr Adolesc Gynecol	2019	33	10.5	–	4—benign cyst 4—serous cystadenoma 6—mature teratoma 2—malignancy 17—not specified	24—CONS 9—OO	13	–
Ollivier et al. [5]	J Pediatr Surgery	2019	54	10	–	11—cystic teratoma 14—follicular cyst 2—serous cystadenoma 27—not specified	50—CONS 4—OO	0	–
Prieto et al. [12]	J Pediatr Surgery	2019	127	13.2	–	113—mass/cyst 14—not specified	105—CONS 22—OO	8	–
Mehmetoğlu [13]	J Med Case Rep	2018	5	6.8	3 R 2 L	3—cyst 2—not specified	2—CONS 2—OO 1 patient had autoamputation	1	–
Hubner et al. [14]	J Pediatr Adolesc Gynecol	2017	139	10.21	–	43—no underlying pathology 70—no functional cyst 20—functional cyst 2—malignancy 4—unknown	103—CONS 36—OO	–	Lap—86 Open—42 Switch to open—11
Comeau et al. [15]	J Pediatr Adolesc Gynecol	2017	11	8.8	6 R 4 L 1 bilat	2—mass 9—no underlying pathology	8 CONS 2 OO 1 partial OO	11	Lap—9 Open—2
Bolli et al. [16]	Medicine (Baltimore)	2017	17	11.0	12 R 5 L	2—mature teratoma 15—not specified	14—CONS 3—OO/SALP	–	Lap—14 Open—3
Bertozzi et al. [17]	J Pediatr Adolesc Gynecol	2017	124 (125 ovaries)	(9.79 ± 3.54)	82 R 41 L 1 synch bilat	59—mass (not specified) 66—no underlying pathology	48—CONS 77—OO	19	Lap—63 Open—51 Switch to open—11
Smorgick et al. [18]	Fertility and Sterility	2016	44	(12.3 ± 4.7)	28 R 16 L	11—simple cyst 3—corpus luteum cyst 6—dermoid cysts 9—paraovarian cyst 2—benign epithelial tumor 1—juvenile granulosa cell tumor 12—not specified	43—CONS 1—OO	7 (2 on the same patient)	Lap—43 Open—1
Oskayli et al. [19]	J Pediatr Adolesc Gynecol	2015	41	(11 ± 3.9)	25 R 17 L	10—simple cyst 6—mature teratoma 1—immature teratoma 1—serous cystadenoma 4—paraovarian cyst 1—hemorrhagic cyst 1—dysgerminoma 17—not specified	26—CONS 16—OO/SALP	12 ipsi 2 contra	Lap—5 Open—36
Santos et al. [20]	J Pediatr Adolesc Gynecol	2015	29	(10.3 ± 4.9)	17 R 12 L	9—functional cyst 4—dermoid cyst 1—mucinous cystadenoma 5—paratubal cyst 4—paraovarian cyst 6—not specified	29—CONS	7 (bilaterally in 1 pt)	Lap—26 Open—2 Reduction through inguinal hernia—1
Ashwal et al. [21]	J Pediatr Adolesc Gynecol	2015	32 (39 ovaries)	9	18 R 21 L	5—simple cyst 1—mature cystic teratoma 33—not specified	37—CONS 2—OO	5	Lap—26 Open—10 Switch to open—3
Agarwal et al. [22]	J Indian Assoc Pediatr Surgery	2014	45	14.3	–	30—simple cyst 7—dermoid 5—paraovarian cyst 3—not specified	40—CONS 5—OO	0	–
Parelkar et al. [23]	J Pediatr Surgery	2014	12 (13 ovaries)	8.84	6 R 7 L	Cases with underlying pathology were excluded	13—CONS	0	Lap—8 Open—2 Switch to open—3
Yildiz et al. [24]	Afr J Paediatr Surg	2014	26	11.42	7 R 19 L	2—simple cyst 2—mature teratoma 17—no underlying pathology 5—no biopsy	15—CONS 11—OO	0	Lap—10 Open—16
Geimanaite et al. [25]	J Pediatr Surgery	2013	50 (53 ovaries)	10.6	34 R 19 L	15—functional cyst 2—serous cystadenoma 6—mature teratoma 1—paraovarian cyst 29—not specified	31—CONS 22—OO	12	Lap—10 Open—42 Switch to open—1
Poonai et al. [26]	Can J Surg	2013	13	12	–	3—hemorrhagic cyst 1—hydrosalpinx 9—not specified	6—CONS (incl. 1 only cystectomy) 6—OO 1—none (spontaneous detorsion)	2	–
Spinelli et al. [3]	Gynecol Endocrinol	2013	29 (30 ovaries)	13.7	21 R 9 L	10—hem. cyst 7—follicular cyst 5—mature teratoma 1—cystadenoma 7—no pathology	14—CONS 16—OO	4	Lap—12 Open—18
Kao et al. [27]	Pediatrics and Neonatology	2012	21	13.62	14 R 7 L	8—teratoma 2—serous cystadenoma 1—follicular cyst 8—simple cyst 1—granulosa cell tumor 1—not specified	7—CONS (incl. 1 patient who didn’t have op) 14—OO/SALP. (incl. partial)	0	Lap—20 Open—1
Total			945 (961 ovaries)		273 R (60.1%) 179 L (39.4%) 2 bilat. (0.44%)	142—normal ovary (15.6%) 252—cyst^d^ (27.8%) 65—mature teratoma^e^ (7.2%) 13—serous/mucinous cystadenoma (1.4%) 3—corpus luteum cyst (0.3%) 2—benign ep. neoplasm (0.2%) 14—hemorrhagic cyst (1.5%) 1—hydrosalpinx (0.1%) 8—malign neoplasm (0.9%) 187—mass, not specified (20.6%) 220—not specified/unknown (24.3%)	708—CONS (73.7%) 250—OO (26.0%) 3—none (0.3%)	106 (14.1%)	Lap—423 (58%) Open—277 (38%) Switch to open—29 (4%)

^a^R: right side; L: left side

^b^CONS: conservative treatment; OO: oophorectomy; SALP: salpingectomy

^c^Lap: laparoscopy

^d^“Cyst” includes: simple cyst, follicular cyst, functional cyst, no functional cyst, paraovarian cyst, paratubal cyst

^e^“Mature teratoma” includes: dermoid cyst, benign teratoma, cystic teratoma

### Systematic literature review

**Fig. 1:**
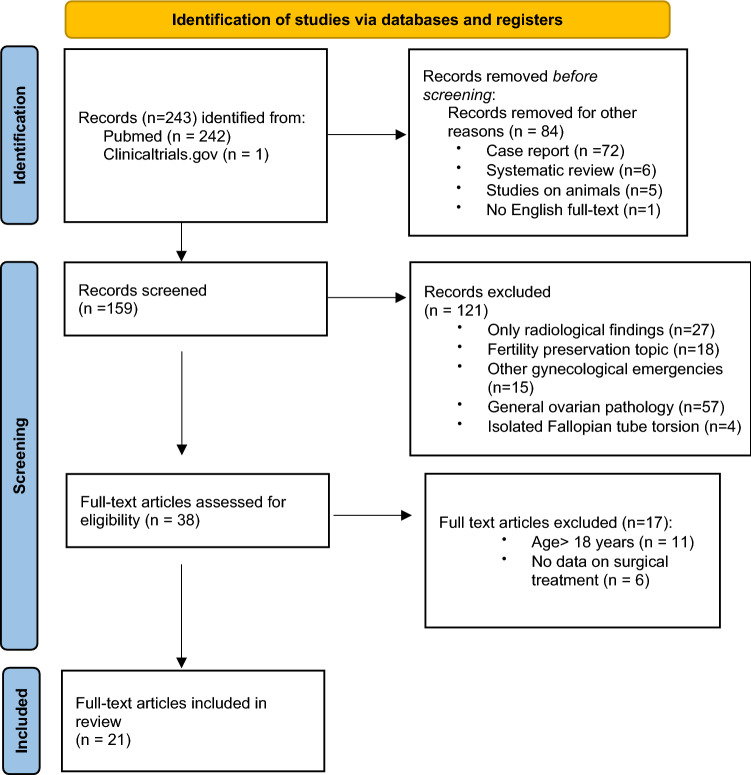
PRISMA 2020^1^ flow diagram for new systematic reviews which included searches of databases and registers only. ^1^The PRISMA 2020 statement: an updated guideline for reporting systematic reviews. *BMJ* 2021;372:n71. For more information, visit: http://www.prisma-statement.org/. Search terms: “Pediatric ovarian torsion”

A total of 243 records were identified from PubMed database and ClinicalTrials.gov. Among these, 84 records were excluded before screening basing on title and abstract, as they did not fulfill inclusion criteria. Therefore, 159 articles remained for further assessment and other 121 records were excluded, as they did not have a pertinent topic. After a full-text review of the 38 remaining eligible articles, 21 articles were included in the systematic review (Fig. 1). The 21 articles [3, 5, 9–27] reported 961 cases of OT on a total of 945 patients. The surgical treatment, whether conservative surgery or oophorectomy, have been reported by all considered articles, with a prevalence of conservative surgery (73.7%); laparoscopy was performed in 423 cases (58%), with a 4% rate of conversion. The histopathology examination was available in 500 cases (55.1%) and showed greater involvement of non-neoplastic lesions (27.8%), including follicular and hemorrhagic cysts, and torsed normal ovaries (15.6%); benign neoplasms accounted for 10.6% of cases and malignant neoplasms were found only in 0.9% of torsions.

The right-left side aspect could be analyzed in a total of 454 cases, with a right majority (60.1%), in addition to two cases (0.4%) of bilateral torsion. Information on the oophoropexy practice, whether performed or not, were reported for a total of 752 cases (78.2%), with 14.1% patients whose ovaries were fixed (Table 2).

**Table 2:** Comparison between anatomo-clinical parameters and type of surgical treatment

Parameter	Conservative	Oophorectomy	*p* value
Age			**0.001**
<12	24	40	
>12	24	9	
Ultrasound diameter			**0.001**
>5 cm	32	48	
<5 cm	16	1	
Hours of pain			0.615
<24	24	22	
>24	24	27	
Pain			**0.002**
Mild/moderate	2	13	
Severe	46	36	
Vomiting			0.337
No	32	37	
Yes	16	12	
Fever			0.414
No	46	45	
Yes	2	4	
Palpable mass			0.068
No	40	33	
Yes	8	16	
Nausea			0.702
No	43	45	
Yes	5	4	
Surgical approach			** <0.001**
Laparoscopy	36	9	
Open	9	28	
Switch	3	12	
Histology			**0.002**
Functional cyst	29	16	
Mature teratoma	5	18	
No underlying patho	2	8	
Malignancy	0	3	
Side of OT			0.366
Right	27	26	
Left	21	21	
Bilateral	0	2	
Tumor markers			0.119
Elevated	1	4	
Not elevated	26	20	

Statistics: absolute frequency

Bold indicates statistical significance values

## Discussion

Our results revealed some important associations between the parameters we investigated. The first one is the one established between pre- and post-menarchal status and the probability of undergoing conservative therapy: girls older than 12 years were, in fact, more likely to be treated with a conservative approach (*p* = 0.001). Nevertheless, this result does not agree with the analyzed literature, in which it was not possible to establish this association [18, 19, 26, 28]. The increased risk of ovarian removal in younger girls may be due to a greater difficulty in expressing symptoms including pain and to the fact that gynecological disease is not often the first line of diagnostic suspicion in such young patients [7].

The second significant association (*p* = 0.001) we observed was between an ovarian size on the preoperative transabdominal US < 5 cm and conservative ovarian-sparing treatment: girls with an ovarian diameter < 5 cm had indeed a bigger chance to undergo conservative therapy, compared to girls with a diameter > 5 cm. The presence of an enlarged ovary (>5 cm) has also been considered as an important predictive factor for the preoperative diagnosis of OT by Melcer et al. [6], and many other studies demonstrated that the risk of OT increases when the ovarian mass is benign and its size is 5 cm or larger, even though children can experience OT with completely normal size ovaries as well [25, 29–32]. Abdomino-pelvic ultrasound is still today the most used and accurate method to investigate the presence of an OT, even though it is operator dependent and has an elevated false-negative rate [33, 34]. Ultrasound findings in OT can include an asymmetric ovarian enlargement, heterogeneous appearance of one ovary due to edema, the presence of a simple or complex adnexal mass, peripherally displaced follicles due to stromal edema from ischemia, medialization of the ovary, displacement of the uterus from the midline, free pelvic fluid, and the whirlpool sign, which consists in the twisting of the ovarian pedicle causing twisting of vessels. Moreover, even though the color Doppler is very helpful to determine the absence/reduction or presence of blood flow, OT cannot be excluded or confirmed with certainty based on this method, because of its low sensitivity (40–73%) [31]. Many authors [24, 35] have in fact described how less than 50% of their patients, confirmed to have OT at surgery, had a decreased or absent flow in the preoperative Doppler ultrasound. In our cohort, we observed a decreased or absent blood flow to the involved ovary in 63.9% of the patients. However, the final diagnosis can be made only by exploratory laparoscopy [3].

As to signs and symptoms, our study showed that the presence of nausea/vomiting, fever, dysuria or a palpable tumor at the clinical examination, which are some of the most frequent observed in OT, was not associated with a conservative treatment. Only the presence of severe abdominal pain can be considered related to conservative surgery (*p* = 0.002): in fact, in our cohort, girls presenting a severe abdominal pain underwent conservative surgery more often than girls with a mild or moderate abdominal pain. This could maybe be explained by the fact that if pain is described as severe, patients tend to report earlier to the medical staff’s attention. Previous studies [2–4, 25, 26, 29, 33, 36] describe abdominal pain as the most frequent symptom of presentation of OT, but we did not find any other multicentric study that correlates the severity of pain with the surgical management.

In our cohort, the duration of pain did not play a role in determining whether the children had a higher or lower chance to be treated conservatively, and this is in accordance with the results deriving from our analysis of the literature [26, 36], except for Rossi et al. [37], who observed a higher rate of oophorectomies in girls who suffered from pain for more than 72 h. Anyway, a rapid diagnosis and intervention are fundamental to prevent an irreversible ovarian damage to maximize the potential salvage of the ovarian tissue, allowing normal progression through puberty and preserving future reproductive capacities [24, 25, 30].

Another association we noted was between laparoscopy and conservative surgery: laparoscopic approach was more likely to be correlated with a conservative treatment (*p* < 0.001), whereas open approach and conversion from laparoscopic to open surgery were more often associated with oophorectomies (*p* < 0.001). This is also confirmed by the analysis of the literature we performed [16, 17, 21, 38]. Laparoscopy is the recommended approach in OT because it reduces diagnostic delays in emergency cases and increases the probability of a conservative management [3, 39]. Even though authors do not yet agree whether the removal of ovarian tissue has an impact on future fertility or not [23, 40], overall studies concur that laparoscopic ovarian-sparing management is the preferred therapeutic option in order to preserve as much ovarian function as possible, and so guarantee an optimal development [22–24, 26, 30].

Regarding the histology, the most frequent associated lesions found in literature [25, 29, 30, 41, 42] include cystic teratomas, follicular or hemorrhagic cyst, paraovarian/paratubal cysts, cystadenoma, and hydrosalpinx. Malignant tumors are, on the contrary, less likely to cause torsion because of their tendency to fix to the adjacent tissues [30]. What we observed in our study is that patients who revealed to present functional cysts were treated in conservative ways, while teratomas and normal torsed ovaries underwent oophorectomy (*p* = 0.002). The analysis of the literature, however, did not demonstrate this relationship [11, 12, 22, 26, 28]. No significant association has, instead, been found between side and type of surgery: right or left side of torsion does not affect the risk to undergo oophorectomy.

Also, regarding the tumor markers, it was not possible to establish a significant association between their elevation and the choice of treatment. The role of tumor markers in the diagnosis and choice of surgical approach of ovarian masses and OT in children is still controversial [43], while an elevation of β-HCG and αFP is highly suggestive of the presence of a trophoblastic tumor, and therefore, requires a deeper investigation, CEA and CA-125 can be elevated in both malignant and benign lesions, and some authors [44, 45] also have described increased tumor markers in children who experienced OT on otherwise normal ovaries. Due to delayed results, tumor marker evaluation has little contribution to decide which surgical strategy should be adopted in an urgent setting when an OT is suspected. Elevated marker values in patients with OT generally go back to normal levels in a month after surgery [43]. Also in our study only one girl, who reported increased levels of αFP, was confirmed to have a malignant neoplasm, while the other four girls (80%) with elevated serum markers were all diagnosed with a mature teratoma. For this reason, high values of CEA and CA-125 should not guide a priori to oophorectomy, and a conservative one is still possible even in case of increased markers. Thus, it has been suggested that, in the presence of positive markers, an intraoperative histopathological analysis should be performed [43].

The reason why we tried to assess potential predictive factors for a conservative surgery in the management of OT is because we believe that this should always be the first choice in the algorithm of therapy. Old concerns for the risk of pulmonary embolism and the risk of malignancy, which led surgeons to treat these girls by practicing oophorectomy in the past, have shown to be groundless, as many authors demonstrated that ovarian functional integrity is not correlated with its ischemic appearance and recovery after detorsion can still occur, even in case of a so-called “black bluish” ovary [39, 46–52]: this is why a conservative management, whenever possible, represents the best option [3, 53]. After this procedure, it is important to observe the possible evolutions through a clinical and US follow-up after 3 months and then every 6/12 months: ovarian tissue can, in fact, recover and reacquire its function, as the presence of follicles will testify, or develop atrophy [49, 51, 54]. If the mass, instead, shows characteristics of malignancy on the ultrasound, such as solidity and heterogeneity, it is necessary, after detorsion, to define its benign or malign nature by performing an intraoperative frozen section [55]. Moreover, we assume that frozen histopathological sections might be useful in case of persistent ischemic normal ovaries following detorsion, to confirm necrosis and this may be especially relevant for legal reasons. In case of malignancy then, an appropriate staging has to be done, and this will also suggest the subsequent steps that need to be adopted [3]. Finally, as to the practice of oophoropexy, which is a surgical technique used to prevent retorsion by fixating the ovary to other structures, like the peritoneum on the pelvic sidewall, the uterosacral ligaments or the round ligament, or by suturing it to the back of the uterus, its utility is still debated [56]. This procedure could be considered in the management of recurrent torsion, which is more likely to occur in case of laxity within the utero-ovarian ligaments, long fallopian tubes, or lack of an adnexal mass: in these patients, oophoropexy has been proposed in order to decrease the likelihood of subsequent retorsion [57]. In this regard, Smorgick et al. [18] noted that recurrent torsion events are more common in those patients who present a first episode of OT in the premenarchal period and have normal ovaries involved, suggesting that in this case oophoropexy could be taken into account by surgeons in the management of this pathology. Others [56] have proposed to perform oophoropexy whenever only one ovary remains due to prior oophorectomy. One disadvantage of oophoropexy is the possible anatomic distortion between the ovary and the fallopian tube, and the consequent interference with the fallopian tube blood supply and function: this may have a negative impact on the fallopian development and fertility [3, 49, 57]. However, this type of fertility reduction has not been described in the medial type of oophoropexy (e.g. utero-ovarian ligament plication), which could thus represent the better option [58, 59]. Moreover, the fixation of the affected ovary may not eliminate the possibility of future episodes of torsion: Geimanaite et al. [25] reported a single case of a patient who underwent oophoropexy but presented then 18 months later with another episode of recurrent torsion. Overall, the efficacy and safety of this procedure are not well established [56] and there is no clear evidence to support oophoropexy in patients who present with a first episode of OT [57].

To our knowledge, this is the first publication describing the association between conservative management of OT and other preoperative and intraoperative parameters of OT in a pediatric cohort of patients treated in Pediatric Surgery Institutions of two different countries. Even acknowledging the limitations of the study, mainly represented by its retrospective nature (with thus some missing or not homogeneously reported data), our results suggest that a post-menarchal age, a pre-operative US ovarian size < 5 cm, the presence of severe abdominal pain, a laparoscopic approach and the presence of a cyst as an underlying pathology are associated with a better chance to undergo conservative ovarian-sparing surgery and thus with a better preservation of future fertility. Considering all, our findings suggest that, regardless of the duration of symptoms, adnexal conservation should always be considered as the first aim of treatment.

## References

[1] Graif M, Itzchak Y (1988). Sonographic evaluation of ovarian torsion in childhood and adolescence. Am J Roentgenol.

[2] Nissen M, Sander V, Rogge P (2020). Neutrophil-to-lymphocyte ratio and platelet-to-lymphocyte ratio may predict pediatric ovarian torsion: a mono-institutional experience and review of literature. J Pediatr Adolesc Gynecol..

[3] Spinelli C, Buti I, Pucci V (2013). Adnexal torsion in children and adolescents: new trends to conservative surgical approach - our experience and review of literature. Gynecol Endocrinol.

[4] Kokoska ER, Keller MS, Weber TR (2000). Acute ovarian torsion in children. Am J Surg.

[5] Ollivier M, Sfar Mohamed S, Tessier B (2019). Torsion of otherwise healthy ovary has a worse prognosis than torsion of pathologic ovary in children. J Pediatr Surg.

[6] Melcer Y, Maymon R, Pekar-Zlotin M (2018). Clinical and sonographic predictors of adnexal torsion in pediatric and adolescent patients. J Pediatr Surg.

[7] Schuh AM, Klein EJ, Allred RJ (2017). Pediatric adnexal torsion: not just a postmenarchal problem. J Emerg Med.

[8] Huchon C, Staraci S, Fauconnier A (2010). Adnexal torsion: a predictive score for pre-operative diagnosis. Hum Reprod.

[9] Julania S, Chown I, Gera S (2020). Management of adnexal torsion in the pediatric and adolescent population at western Australia's single tertiary children's hospital over the last 10 years: retrospective study. J Minim Invasive Gynecol.

[10] Geimanaite L, Trainavicius K (2019). Pediatric ovarian torsion: follow- up after preservation of ovarian tissue. J Pediatr Surg.

[11] Tasset J, Rosen MW, Bell S (2019). Ovarian torsion in premenarchal girls. J Pediatr Adolesc Gynecol.

[12] Prieto JM, Kling KM, Ignacio RC (2019). Premenarchal patients present differently: a twist on the typical patient presenting with ovarian torsion. J Pediatr Surg.

[13] Mehmetoğlu F (2018). How can the risk of ovarian retorsion be reduced?. J Med Case Rep.

[14] Hubner N, Langer JC, Kives S, Allen LM (2017). Evolution in the management of pediatric and adolescent ovarian torsion as a result of quality improvement measures. J Pediatr Adolesc Gynecol.

[15] Comeau IM, Hubner N, Kives SL, Allen LM (2017). Rates and technique for oophoropexy in pediatric ovarian torsion: a single-institution case series. J Pediatr Adolesc Gynecol.

[16] Bolli P, Schädelin S, Holland-Cunz S, Zimmermann P (2017). Ovarian torsion in children: Development of a predictive score. Medicine (Baltimore).

[17] Bertozzi M, Esposito C, Vella C (2017). Pediatric ovarian torsion and its recurrence: a multicenter study. J Pediatr Adolesc Gynecol.

[18] Smorgick N, Melcer Y, Sarig-Meth T (2016). High risk of recurrent torsion in premenarchal girls with torsion of normal adnexa. Fertil Steril.

[19] Oskaylı MÇ, Durakbaşa ÇU, Maşrabacı K (2015). Surgical approach to ovarian torsion in children. J Pediatr Adolesc Gynecol.

[20] Santos XM, Cass DL, Dietrich JE (2015). Outcome following detorsion of torsed adnexa in children. J Pediatr Adolesc Gynecol.

[21] Ashwal E, Krissi H, Hiersch L (2015). Presentation, diagnosis, and treatment of ovarian torsion in premenarchal girls. J Pediatr Adolesc Gynecol.

[22] Agarwal P, Agarwal P, Bagdi R (2014). Ovarian preservation in children for adnexal pathology, current trends in laparoscopic management and our experience. J Indian Assoc Pediatr Surg.

[23] Parelkar SV, Mundada D, Sanghvi BV (2014). Should the ovary always be conserved in torsion? A tertiary care institute experience. J Pediatr Surg.

[24] Yildiz A, Erginel B, Akin M (2014). A retrospective review of the adnexal outcome after detorsion in premenarchal girls. Afr J Paediatr Surg.

[25] Geimanaite L, Trainavicius K (2013). Ovarian torsion in children: management and outcomes. J Pediatr Surg.

[26] Poonai N, Poonai C, Lim R (2013). Pediatric ovarian torsion: case series and review of the literature. Can J Surg.

[27] Kao JK, Chiu CC, Wang PY (2012). Pediatric ovarian torsion in a medical center in Taiwan: case analysis. Pediatr Neonatol.

[28] Pathak IS, Jurak J, Mulla ZD (2018). Predictors of oophorectomy in girls hospitalized in Texas with ovarian torsion. Hosp Pediatr.

[29] Cass DL (2005). Ovarian torsion. Semin Pediatr Surg.

[30] Childress KJ, Dietrich JE (2017). Pediatric ovarian torsion. Surg Clin North Am.

[31] Lourenco AP, Swenson D, Tubbs RJ (2014). Ovarian and tubal torsion: imaging findings on US, CT, and MRI. Emerg Radiol.

[32] Gerscovich EO, Corwin MT, Sekhon S (2014). Sonographic appearance of adnexal torsion, correlation with other imaging modalities, and clinical history. Ultrasound Q.

[33] Oltmann SC, Fischer A, Barber R (2009). Cannot exclude torsion - a 15-year review. J Pediatr Surg.

[34] Servaes S, Zurakowski D, Laufer MR (2007). Sonographic findings of ovarian torsion in children. Pediatr Radiol.

[35] Stark JE, Siegel MJ (1994). Ovarian torsion in prepubertal and pubertal girls: Sonographic findings. AJR.

[36] Piper HG, Oltmann SC, Xu L (2012). Ovarian torsion: diagnosis of inclusion mandates earlier intervention. J Pediatr Surg.

[37] Rossi BV, Ference EH, Zurakowski D (2012). The clinical presentation and surgical management of adnexal torsion in the pediatric and adolescent population. J Pediatr Adolesc Gynecol.

[38] Walker SK, Lal DR, Boyd KP (2018). Management of pediatric ovarian torsion: evidence of follicular development after ovarian preservation. Surgery.

[39] Galinier P, Carfagna L, Delsol M (2009). Ovarian torsion. Management and ovarian prognosis: a report of 45 cases. J Pediatr Surg..

[40] Bellati F, Ruscito I, Gasparri ML (2014). Effects of unilateral ovariectomy on female fertility outcome. Arch Gynecol Obstet.

[41] Spinelli C, Di Giacomo M, Cei M (2009). Functional ovarian lesions in children and adolescents: when to remove them. Gynecol Endocrinol.

[42] Spinelli C, Di Giacomo M, Mucci N, Massart F (2009). Hemorrhagic corpus luteum cysts: an unusual problem for pediatric surgeons. J Pediatr Adolesc Gynecol.

[43] Spinelli C, Pucci V, Buti I (2012). The role of tumor markers in the surgical approach of ovarian masses in pediatric age: a 10-year study and a literature review. Ann Surg Oncol.

[44] Savic D, Stankovic ZB, Djukic M (2008). Torsion of malignant ovarian tumors in childhood and adolescence. J Pediatr Endocrinol Metab.

[45] McCarthy JD, Erickson KM, Smith YR (2010). Premenarchal ovarian torsion and elevated Ca-125. J Pediatr Adolesc Gynecol.

[46] Taskin O, Birincioglu M, Aydin A (1998). The effects of twisted ischaemic adnexa managed by detorsion on ovarian viability and histology: an ischaemia-reperfusion rodent model. Hum Reprod.

[47] Adeyemi-Fowode O, Lin EG, Syed F, Sangi-Haghpeykar H, Zhu H, Dietrich JE (2019). Adnexal torsion in children and adolescents: a retrospective review of 245 cases at a single institution. J Pediatr Adolesc Gynecol.

[48] Guthrie BD, Adler MD, Powell EC (2010). Incidence and trends of pediatric ovarian torsion hospitalizations in the United States, 2000–2006. Pediatrics.

[49] Celik A, Ergün O, Aldemir H (2005). Long-term results of conservative management of adnexal torsion in children. J Pediatr Surg.

[50] Göçmen A, Karaca M, Sari A (2008). Conservative laparoscopic approach to adnexal torsion. Arch Gynecol Obstet.

[51] Templeman C, Hertweck SP, Fallat ME (2000). The clinical course of unresected ovarian torsion. J Pediatr Surg.

[52] Aziz D, Davis V, Allen L (2004). Ovarian torsion in children: is oophorectomy necessary?. J Pediatr Surg.

[53] Spinelli C, Piscioneri J, Strambi S (2015). Adnexal torsion in adolescents: update and review of the literature. Curr Opin Obstet Gynecol.

[54] Beaunoyer M, Chapdelaine J, Bouchard S (2004). Asynchronous bilateral ovarian torsion. J Pediatr Surg.

[55] Suprasert P, Khunamornpong S, Phusong A (2008). Accuracy of intra-operative frozen sections in the diagnosis of ovarian masses. Asian Pac J Cancer Prev.

[56] Childress KJ, Dietrich JE (2017). Pediatric ovarian torsion. Surg Clin N Am.

[57] Dasgupta R, Renaud E, Goldin AB (2018). Ovarian torsion in pediatric and adolescent patients: a systematic review. J Pediatr Surg.

[58] Abes M, Sarihan H (2004). Oophoropexy in children with ovarian torsion. Eur J Pediatr Surg.

[59] Simsek E, Kilicdag E, Kalayci H (2013). Repeated ovariopexy failure in recurrent adnexal torsion: combined approach and review of the literature. Eur J Obstet Gynecol Reprod Biol.

